# Proteinuria is a key to suspect autoimmune nodopathies

**DOI:** 10.1111/ene.16406

**Published:** 2024-07-09

**Authors:** Kei Funakoshi, Norito Kokubun, Keisuke Suzuki, Nobuhiro Yuki

**Affiliations:** ^1^ Department of Neurology Dokkyo Medical University Tochigi Japan; ^2^ Department of Neurology Takai Hospital Nara Japan

**Keywords:** autoimmune nodopathy, chronic inflammatory demyelinating polyneuropathy, contactin 1, membranous nephropathy, neurofascin 155

## Abstract

**Background and purpose:**

Reports of patients who have autoimmune nodopathies concurrent with nephrotic syndrome are increasing. We investigated whether proteinuria could be a biomarker of autoimmune nodopathies.

**Methods:**

Qualitative urinalysis results were retrospectively obtained from 69 patients who were diagnosed with chronic inflammatory demyelinating polyneuropathy (CIDP) at a hospital in Japan. Proteinuria was graded as mild to severe (i.e., mild, 30–99; moderate, 100–299; severe, 300 mg/dL or more) according to the results of the urine dipstick test. Autoantibodies against the paranodal proteins contactin 1 (CNTN1), neurofascin 155 (NF155), and contactin‐associated protein 1 (Caspr1) and the nodal protein neurofascin 186 (NF186) were measured, and the predominant IgG subclass was determined by enzyme‐linked immunosorbent assay in sera from the 69 patients.

**Results:**

Four patients (6%), five patients (7%), and one (1%) patient were positive for anti‐CNTN1, anti‐NF155, and anti‐Caspr1 IgG4 antibodies, respectively. No patients had IgG4 antibodies against NF186. Proteinuria of mild or greater levels was found in three patients with anti‐CNTN1 IgG4 and two patients with anti‐NF155 IgG4 antibodies. The autoantibody‐positive patients more frequently had proteinuria of mild or greater levels than the seronegative patients (*p* = 0.01).

**Conclusions:**

Proteinuria is a possible biomarker of autoimmune nodopathies associated with autoantibodies targeting CNTN1 or NF155. Urinalysis results should be carefully checked for quick differentiation of autoimmune nodopathies from CIDP. Patients who present with nephrotic syndrome should be tested for anti‐CNTN1 IgG4 antibodies, and patients who exhibit mild proteinuria should be tested for anti‐NF155 IgG4 antibodies.

## INTRODUCTION

Chronic inflammatory demyelinating polyneuropathy (CIDP), a group of chronic immune‐mediated demyelinating neuropathies, has variable clinical presentations and treatment responses, suggesting an underlying heterogeneous pathophysiology [1]. Autoimmune nodopathies have been distinguished from CIDP in recent years because patients with autoantibodies against nodal and paranodal molecules, such as neurofascin 155 (NF155), contactin 1 (CNTN1), contactin‐associated protein 1 (Caspr1), and neurofascin 186 (NF186), have specific clinical presentations [2]. However, clinical or neurophysiological identification of autoimmune nodopathies is often impossible without measuring autoantibodies. Moreover, only a few laboratories can perform autoantibody testing, and it takes several weeks or more to obtain results. Easily detected biomarkers that can identify patients who require autoantibody testing as well as select a more appropriate choice of first‐line treatment other than intravenous immunoglobulin (IVIg) are needed.

Membranous nephropathy, a major cause of nephrotic syndrome in adults, is rarely accompanied by CIDP [3]. In contrast, reports of patients with both autoimmune nodopathies and membranous nephropathy associated with autoantibodies targeting CNTN1 are increasing [4, 5, 6, 7, 8]. To date, CNTN1 has been recognized as a novel target in primary membranous nephropathy [9]. Most of the reported patients showed heavy proteinuria or significant nephrotic syndrome and underwent renal biopsies for diagnosis. Although the importance of both light and heavy proteinuria in disease pathophysiology might be equal, patients with asymptomatic or mild proteinuria are likely often overlooked. To elucidate the correct pathophysiology of these conditions, detection of mild proteinuria is essential.

Although high prevalence of nephrotic syndrome in autoimmune nodopathies associated with anti‐CNTN1 IgG4 [5, 7, 10, 11], anti‐pan‐neurofascin IgG1 [12], or anti‐NF186 IgG [13] has increasingly been emphasized, routine urinalysis, which can be performed at every hospital, is not recommended in the diagnostic guidelines [2, 14]. Therefore, in our study, we tested whether proteinuria can be a biomarker of autoimmune nodopathy associated with anti‐CNTN1 IgG4 antibodies in patients with suspected CIDP at a single hospital in Japan. Unexpectedly, we also found that proteinuria could be a possible biomarker of autoimmune nodopathy associated with anti‐NF155 IgG4 antibodies.

## METHODS

### Patients

The medical records of 71 consecutive patients who visited Dokkyo Medical University Hospital between November 1997 and January 2023 and fulfilled the European Federation of Neurological Societies/Peripheral Nerve Society criteria for CIDP [14] were reviewed retrospectively. All the patients underwent systematic electrodiagnostic tests as described elsewhere [15]. Results of urinalysis and blood chemistry tests were collected from 69 of the patients included in the study at their first visit. Of the 69 patients, 21 had been included in our previous studies [16, 17]. Blood or urinary test results suggested neither urinary tract infection nor other renal diseases in our patients.

The patients were classified into subtypes according to the existing criteria [18] as follows. The distal variant was defined by distal leg‐dominant symmetric muscle weakness with fully preserved proximal limb muscles; the multifocal variant was defined by muscle weakness with sensory symptoms in a multifocal distribution; the sensory‐predominant variant was defined by sensory symptoms without muscle weakness but with demyelinating motor conduction abnormalities; and the motor‐predominant variant was defined by muscle weakness without sensory symptoms but with sensory nerve conduction abnormalities. Treatment response was defined as an improvement in muscle weakness (increase of 4 or more points on the Medical Research Council sum score) within 4 weeks after any of the treatments.

### Urinalysis

A spot urine dipstick test was used to diagnose proteinuria. The grades of proteinuria were as follows: −, not detected; 1+, 30–99 mg/dL; 2+, 100–299 mg/dL; 3+, 300–999 mg/dL; and 4+, 1000 mg/dL or more. A score of 1+ was defined as mild, 2+ as moderate, and 3+ or more as severe proteinuria. The global diagnostic criteria for nephrotic syndrome included persistent proteinuria of 3.5 g/day or more or a random spot urine protein–creatinine ratio of 3.5 g/gCr or more, hypoalbuminemia (<3.0 g/dL), and edema [19].

### Serological studies

Sera from patients were stored at −80°C in a deep freezer used for autoantibody assays. IgG subclass (IgG1 and IgG3 [Thermo Fisher Scientific, Waltham, MA, USA], IgG4 [SouthernBiotech, Birmingham, AL, USA]) antibodies against CNTN1 (Sino Biological, Beijing, China), NF155 and NF186 (OriGene Technologies, Rockville, MD, USA), and Caspr1 (R&D Systems, Minneapolis, MN, USA) were tested by enzyme‐linked immunosorbent assays as described elsewhere, with minor modifications [17].

### Standard protocol approvals, registrations, and patient consents

This study was performed in accordance with the guidelines stipulated in the Declaration of Helsinki and Ethical Guidelines for Medical and Biological Research Involving Human Subjects by the Japanese government and was approved by the ethics review committee of Dokkyo Medical University (approval number R‐41‐5J).

### Statistical analyses

Differences in proportions were examined by Fisher exact test. Student *t*‐test or Welch *t*‐test was used for normally distributed continuous variables, and Mann–Whitney *U‐*test was used for nonnormally distributed variables. A difference was considered significant at a *p*‐value less than 0.05. Analyses were performed with SPSS 25J software (SPSS, Chicago, IL, USA).

## RESULTS

### IgG subclasses of antibodies to paranodal or nodal proteins

Table 1 shows the clinical features of the patients. Of the 69 patients, four (6%) were positive for anti‐CNTN1 antibodies, five (7%) were positive for anti‐NF155 antibodies, and one (1%) was positive for anti‐Caspr1 IgG4 antibodies. Among the four patients positive for anti‐CNTN1 IgG4, the IgG1 subclass was weakly positive in three patients. In the patient positive for anti‐Caspr1 IgG4, the IgG1 subclass was also detected weakly. The five patients positive for anti‐NF155 IgG4 did not have autoantibodies of the IgG1 subclass. No patients had both anti‐NF155 and anti‐NF186 autoantibodies concurrently, suggesting that the anti‐NF155 IgG4 antibodies did not react with NF186. The patients who did not have the IgG4 autoantibodies described above were considered “seronegative.”

**TABLE 1:** Clinical profiles of patients with chronic inflammatory demyelinating polyneuropathy.

Characteristics	Total	IgG4 antibodies to				*p* values against seronegative	
		CNTN1	NF155	Caspr1	Seronegative	Anti‐CNTN1 IgG4	Anti‐NF155 IgG4
Patients, *n* (%)	69 (100)	4 (6)	5 (7)	1 (1)	59 (86)		
Gender, male/female	48/21	4/0	4/1	1/0	39/20	0.21	0.47
Age at onset (years, median [range])	54 [4–86]	63 [35–69]	34 [27–45]	62	55 [4–86]	0.40	0.02
Time to first visit (months, median [range])	5 [0.2–348] (*n* = 67)	4 [1–12]	10 [6–204]	2	5 [0.2–348] (*n* = 57)	0.74	0.09
Clinical phenotype, *n* (%)							
Typical	50 (72)	4 (100)	2 (40)	1 (100)	43 (73)	0.30	0.15
Multifocal	7 (10)	0 (0)	0 (0)	0 (0)	7 (12)	0.62	0.55
Motor‐predominant	5 (7)	0 (0)	0 (0)	0 (0)	5 (8)	0.71	0.66
Distal	2 (3)	0 (0)	1 (20)	0 (0)	1 (2)	0.94	0.15
Sensory‐predominant	5 (7)	0 (0)	2 (40)	0 (0)	3 (5)	0.82	0.04
Neurological signs during illness, *n* (%)							
Cranial nerve involvement	10 (14)	2 (50)	1 (20)	0 (0)	7 (12)	0.09	0.50
Muscle atrophy	8 (12)	2 (50)	0 (0)	0 (0)	6 (10)	0.08	0.60
Tremor	12 (17)	2 (50)	4 (80)	1 (100)	5 (8)	0.06	<0.001
Sensory ataxia	6 (10)	2 (50)	3 (60)	0 (0)	1 (2)	0.009	<0.001
Hughes grade at diagnosis, *n* (%)							
Grade 2 or less	22 (32)	1 (25)	2 (40)	0 (0)	19 (32)	0.62	0.53
Grade 3 or more	47 (68)	3 (75)	3 (60)	1 (100)	40 (68)	0.62	0.81
Grade 4 or more	31 (45)	2 (50)	1 (20)	1 (100)	27 (46)	0.63	0.27
Grade 5 or more	3 (4)	0 (0)	0 (0)	0 (0)	3 (5)	0.82	0.78
Cerebrospinal fluid	*n* = 64	*n* = 4	*n* = 4	*n* = 1	*n* = 55		
Protein (g/L, median [range])	0.93 [0.13–4.55]	1.93 [1.42–3.85]	2.15 [1.59–3.48]	4.55	0.85 [0.13–3.59]	0.03	0.01
Albuminocytological dissociation, *n* (%)	50 (78)	4 (100)	4 (100)	1 (100)	41 (75)	0.33	0.33
Clinical course, *n* (%)							
Relapsing–remitting	36 (52)	0 (0)	4 (80)	1 (100)	31 (53)	0.06	0.24
Chronic progressive	17 (25)	2 (50)	1 (20)	0 (0)	14 (24)	0.27	0.67
Monophasic	16 (23)	2 (50)	0 (0)	0 (0)	14 (24)	0.27	0.28
Treatment response: responders/ patients applied treatment, *n* (%)							
Intravenous immunoglobulin	50/64 (78)	1/4 (25)	2/5 (40)	0/1 (0)	46/54 (85)	0.02	0.04
Corticosteroids	37/57 (65)	2/4 (50)	4/5 (80)	1/1 (100)	30/47 (64)	0.48	0.43
Plasmapheresis	7/14 (50)	0/1 (0)	1/2 (50)	0/1 (0)	6/10 (60)	0.46	0.68

Abbreviations: Caspr1, contactin‐associated protein 1; CNTN1, contactin 1; NF155, neurofascin 155.

### Anti‐CNTN1 IgG4‐positive patients

Patients who carried anti‐CNTN1 IgG4 antibodies more frequently exhibited sensory ataxia (*p* = 0.009). All four patients had the typical CIDP phenotype. The cerebrospinal fluid protein level was significantly greater in patients who harbored anti‐CNTN1 antibodies than in seronegative patients (*p* = 0.03). The four patients received both IVIg and corticosteroids. One (25%) of the patients responded to IVIg, which was less effective than it was for the seronegative patients (*p* = 0.02), and two (50%) patients responded to corticosteroids. Patient 1, as shown in Table 2, was described elsewhere in detail [3, 16]. Three of four (75%) patients who had anti‐CNTN1 antibodies showed severe proteinuria, two of whom were histologically diagnosed as having membranous nephropathy.

**TABLE 2:** Anti‐CNTN1 IgG4‐positive autoimmune nodopathy concurrent with nephrotic syndrome: Our patients and a literature search.

Patient no.	Age at onset, years/gender	Modified Rankin Scale	Phenotype	Sequence of symptoms manifestation	Sensory ataxia	Deep sense impairment	Tremor	CSF protein, g/L	Serum albumin, g/dL	Urine protein, g/day	Treatment response to CIDP	Treatment response to nephrotic syndrome
1 (current series)	36/male	4	Typical	Neurological symptoms preceded renal one	+	+	+	6.35	2.9	2.57	IVIg, corticosteroids, PE, cyclosporin or tacrolimus, ineffective	IVIg, corticosteroids, PE, cyclosporin or tacrolimus, partially effective
2 (current series)	69/male	4	Typical	Neurological symptoms preceded renal one	+	+	−	3.85	3.1	5.02	Corticosteroids, IVIg, PE, cyclophosphamide, ineffective	Corticosteroids, IVIg, PE, cyclophosphamide, ineffective
3 (current series)	68/male	1	Typical	Neurological symptoms preceded renal one	−	+	−	1.42	2.1	1.34	IVIg, ineffective; corticosteroids, effective	Corticosteroids, ineffective
4 (Doppler et al. [4])	48/male	5	Typical	Concurrent	ND	Severe sensory disturbances	+	2.04	ND	ND	IVIg, initially effective; corticosteroids, transiently effective; PE, effective	Complete recovery (treatment efficacy was not well documented)
5 (Hashimoto et al. [5])	78/female	4	Typical	Concurrent	+	+	−	0.61	0.7	4.1	IVIg, effective; corticosteroids, ineffective	Corticosteroids, effective
6 (Taieb et al. [10])	75/male	4	Typical	Neurological symptoms preceded renal one	+	+	ND	1.5	2.2	5.4	Corticosteroids, PE, rituximab, ineffective	Corticosteroids, effective
7 (Cortese et al. [6])	58/male	4	Typical	Concurrent	+	+	−	ND	ND	ND	IVIg, ineffective; corticosteroids, transiently effective; PE, cyclophosphamide, effective	Cyclophosphamide, effective
8 (Nazarali et al. [11])	43/male	4	Typical	Neurological symptoms preceded renal one	ND	+	ND	ND	1.5	22	IVIg, corticosteroids, effective	IVIg, ineffective; corticosteroids, cyclosporin, effective

Abbreviations: CIDP, chronic inflammatory demyelinating polyneuropathy; CNTN1, contactin 1; CSF, cerebrospinal fluid; IVIg, intravenous immunoglobulin; ND, not documented; PE, plasma exchange.

### Anti‐NF155 IgG4‐positive patients

The patients who had anti‐NF155 IgG4 antibodies exhibited the sensory‐predominant phenotype (2/5, 40%) more frequently than seronegative patients (3/59, 5%; *p* = 0.04). Two patients had the typical phenotype, and one patient had the distal phenotype. Neither the male‐to‐female ratio nor the disease severity differed between the anti‐NF155‐positive and seronegative patients. The anti‐NF155‐positive patients had an earlier disease onset than the seronegative patients (*p* = 0.02). Patients exhibited finger tremors (*p* < 0.001) and sensory ataxia (*p* < 0.001) more frequently than seronegative patients. The cerebrospinal fluid protein level was greater in the anti‐NF155‐positive patients than in the seronegative patients (*p* = 0.01). Two of the five patients showed a relatively favorable response to IVIg (40%), but less frequently than seronegative patients (*p* = 0.04), and four responded to corticosteroids (80%).

### Urine analysis

As shown in Table 3, proteinuria of mild or greater severity was found in five of nine patients who carried IgG4 antibodies against CNTN1 or NF155 (seropositive patients) and nine of 59 seronegative patients (odds ratio [OR] = 6.9, 95% confidence interval [CI] = 1.7–29, *p* = 0.01). The patient who harbored the anti‐Caspr1 IgG4 antibodies did not have proteinuria. Moderate or greater proteinuria was found in three of nine seropositive patients and four of 59 seronegative patients (OR = 6.9, 95% CI = 1.4–35, *p* = 0.04). Severe proteinuria was found in three of nine seropositive patients and zero of 59 seronegative patients (OR = 64, 95% CI = 5.0–735, *p* < 0.001). Patients who carried anti‐CNTN1 IgG4 antibodies had proteinuria more frequently than those who did not. In contrast, mild or greater proteinuria was found in two of five (40%) patients who had anti‐NF155 IgG4 antibodies and nine of 59 (15%) seronegative patients (*p* = 0.20). Moderate or greater proteinuria was found in zero of five anti‐NF155‐positive patients and four of 59 (7%) seronegative patients (*p* = 0.72). No patients in the anti‐NF155‐positive or seronegative groups had severe proteinuria. Urine glucose was detected in zero of nine seropositive patients and seven of 59 (12%) seronegative patients, but the difference was not significant (OR = 0.37, 95% CI = 0.04–4.2, *p* = 0.32).

**TABLE 3:** Odds ratios of the grade of proteinuria for autoimmune nodopathies.

Proteinuria	*n*	OR (95% CI)	*p* values
Anti‐CNTN1 or anti‐NF155 vs. seronegative			
Mild or more	5/9 vs. 9/59	6.9 (1.7–29)	0.01
Moderate or more	3/9 vs. 4/59	6.9 (1.4–35)	0.04
Severe	3/9 vs. 0/59	64 (5.0–735)	<0.001
Anti‐CNTN1 positive vs. anti‐CNTN1 negative			
Mild or more	3/4 vs. 11/65	15 (1.9–111)	0.02
Moderate or more	3/4 vs. 4/65	46 (5.0–389)	0.003
Severe	3/4 vs. 0/65	306 (16–4484)	<0.001

*Note*: Mild: 30–99 mg/dL, moderate: 100–299 mg/dL, severe: 300 mg/dL or more. “Seronegative” means that neither anti‐CNTN1, anti‐contactin‐associated protein 1, nor anti‐NF155 IgG4 antibodies were detected.

Abbreviations: CI, confidence interval; CNTN1, contactin 1; NF155, neurofascin 155; OR, odds ratio.

## DISCUSSION

In our cohort of 69 patients who were diagnosed with CIDP, the typical clinical phenotype was predominant (72%), followed by the multifocal variant (10%), which was consistent with the findings of previous studies [18, 20]. In our cohort, 10 (14%) patients had autoimmune nodopathies associated with IgG4 autoantibodies targeting CNTN1 (four patients, 6%), NF155 (five patients, 7%), and Caspr1 (one patient, 1%). Several studies have shown that anti‐CNTN1 IgG4 antibody‐associated nodopathy accounted for 1%–8% of CIDP cohorts [4, 6, 8, 12, 16, 21, 22], whereas anti‐NF155 IgG4 antibody‐associated nodopathy accounted for 1%–18% [6, 8, 12, 17, 22, 23, 24, 25, 26, 27, 28], and anti‐Caspr1 IgG4 antibody‐associated nodopathy accounted for 0.2%–0.9% [4, 6, 7, 8, 16, 21, 22]. Our patients with anti‐CNTN1 nodopathy had significantly more frequent sensory ataxia and a greater incidence of poor response to IVIg, which was consistent with earlier reports [4, 6, 16, 21]. Our patients with anti‐NF155 nodopathy had a significantly earlier onset of disease, more frequent tremors and sensory ataxia, and higher cerebrospinal fluid protein concentrations and showed partial response to IVIg compared to those with seronegative CIDP, which are reported characteristics of anti‐NF155 nodopathy [17, 23, 24, 28] (Table 1). All of the anti‐CNTN1 nodopathy patients and 40% of the anti‐NF155 nodopathy patients in our cohort exhibited the clinically typical CIDP phenotype. Three of four (75%) patients with anti‐CNTN1 nodopathy had severe proteinuria, two of whom were histologically diagnosed as having membranous nephropathy.

Previous reports have described that nephrotic syndrome is rarely accompanied by CIDP in patients who do not carry autoantibodies against nodal and paranodal proteins (0/100, 0% [8]; 5/147, 3% [7]). In contrast, nephrotic syndrome frequently co‐occurred with autoimmune nodopathies in patients harboring autoantibodies targeting nodal and paranodal proteins, especially CNTN1 (up to 8/10, 80%; Table 4) [4, 6, 7, 8, 22]. Membranous nephropathy was histologically identified in 15 patients who had both nephrotic syndrome and anti‐CNTN1 IgG antibodies [4, 7, 8]. Moreover, case series and case reports have shown a high incidence of membranous nephropathy in patients with anti‐CNTN1 nodopathy [5, 7, 10, 11]. IgG4 was the predominant subclass in 23 of 24 patients (96%) who harbored anti‐CNTN1 IgG antibodies [4, 5, 6, 7, 8, 10, 11]. Renal biopsy revealed IgG4 deposition on the glomerular basement membrane [5, 7, 8, 10]. Clinical information was available for 21 patients who had anti‐CNTN1 nodopathy concurrent with membranous nephropathy [4, 5, 6, 7, 10, 11]. Except for leg edema (10/18, 56%), the clinical characteristics were indistinguishable from those of anti‐CNTN1 nodopathy without concurrent membranous nephropathy [4, 5, 6, 7, 10, 11]. Two of five (40%) patients who had IgG3 and IgG4 antibodies against the nodal proteins NF140 and NF186, isoforms of NF155, presented with nephrotic syndrome, the histology of which was focal segmental glomerulosclerosis [22]. Subsequently, patients with autoimmune nodopathy who had anti‐NF186 IgG antibodies and focal segmental glomerulosclerosis were also identified (IgG subclass was not shown) [13]. Three of the eight (38%) patients who harbored IgG1 antibodies against NF186, NF140, and NF155 (pan‐neurofascin) had nephrotic syndrome, the histological findings of which were not reported [12]. In contrast, none of the patients with anti‐NF155 nodopathy presented with nephrotic syndrome [6, 8, 12, 22]. Interestingly, two of five (40%) patients with NF155 nodopathy in our study had mild proteinuria without apparent symptoms of nephropathy.

**TABLE 4:** Autoimmune nodopathies concomitant with nephropathy.

Autoantibodies against	Doppler et al. [4]	Delmont et al. [22]	Cortese et al. [6]	Delmont et al. [8]	Fehmi et al. [7]
CNTN1	1/4 (25%)	0/2 (0%)	1/3 (33%)	6/10 (60%)	8/10 (80%)
NF155	NE	0/9 (0%)	0/9 (0%)	0/15 (0%)	0/13 (0%)
NF140 and NF186	NE	2/5 (40%)	0/1 (0%)	0	NE
NF186	NE	NE	NE	NE	0/1 (0%)
Caspr1	NE	0/2 (0%)	0/6 (0%)	0/2 (0%)	0/5 (0%)
CNTN1/Caspr1 complex	NE	NE	NE	NE	0/1 (0%)
NF140, NF186, and NF155	NE	NE	NE	NE	2/6 (33%)

Abbreviations: Caspr1, contactin‐associated protein 1; CNTN1, contactin 1; NE, not examined; NF, neurofascin.

Several nodal/paranodal molecules are recognized as target antigens for autoimmune nodopathies; for example, NF155 on the terminal loop of myelin and CNTN1 and Caspr1 on the axolemma form septatelike junctions between myelin loops and the axolemma at the paranodes [29]. NF186, expressed on the axolemma at the nodes of Ranvier, forms a complex with gliomedin and neuronal cell adhesion molecule on Schwann cell microvilli and regulates sodium channel clustering on the axolemma at the nodes of Ranvier [30]. These paranodal and nodal molecules play crucial roles in the generation of saltatory nerve transmission of myelinated nerve fibers [29, 31]. Detachment of the terminal loops of myelin and the axolemma at the paranodes was identified in patients with anti‐CNTN1 and anti‐NF155 nodopathies [32, 33]. These conditions might disrupt or alter the distributions of both sodium and potassium ion channel clusters at nodes and juxtaparanodes and impair saltatory conduction via a reduction in sodium ion influx and hyperpolarization of membrane potential [34]. Autoantibodies against phospholipase A2 receptor (PLA2R) and thrombospondin type 1 domain‐containing 7A (THSD7A) are pathogenic autoantibodies found in primary membranous nephropathy, and their predominant IgG subclass is IgG4 [35, 36]. PLA2R and THSD7A are expressed on the cell surface of podocytes [35, 36]. In membranous nephropathy, IgG4 antibodies against PLA2R or THSD7A penetrate the glomerular loop and glomerular basal membrane and induce the cytotoxicity of podocytes and leakage of proteins from vessels to renal tubules [35]. Contactin is also expressed in the kidney as well as the nerves [37], although its localization in the kidney has yet to be determined. Four of 295 (1.4%) patients with isolated membranous nephropathy without neuropathy had anti‐CNTN1 IgG4 antibodies [7], suggesting that contactin was expressed on the podocyte, glomerular loop, or glomerular basal membrane. NF186 is also likely expressed on glomerular podocytes as well as the nodal axolemma [38]. It is unclear why patients with anti‐NF155 nodopathy in our cohort exhibited mild proteinuria, because NF155 expression has yet to be detected in the kidney, and our results indicate that the anti‐NF155 antibodies do not react with NF186.

Some patients with CIDP mimics can also experience proteinuria. The differential diagnosis, which is listed in the revised CIDP guidelines by the European Academy of Neurology/Peripheral Nerve Society, includes conditions that can cause proteinuria [2]. Diabetic polyneuropathy and uremic neuropathy often present with proteinuria, depending on the severity or stage of the primary disease. Patients with amyloid light chain amyloidosis or transthyretin familial amyloid polyneuropathy can present with amyloid nephropathy [39]. Multiple myeloma or osteosclerotic myeloma can cause light chain cast nephropathy or cryoglobulinemic glomerulonephritis [40]. Anti‐neutrophil cytoplasmic antibody‐associated vasculitis is often associated with glomerulonephritis [41]. Moreover, two of five patients who harbored anti‐NF140 and NF186 IgG antibodies had nephrotic syndrome caused by focal segmental glomerulosclerosis [22], and their electrodiagnosis was an axonal neuropathy. Therefore, patients with suspected CIDP, with or without demyelinating neurophysiology, should be screened for proteinuria, followed by anti‐nodal/paranodal autoantibody measurement.

Our study has several limitations. First, there was a small number of anti‐NF155‐ and anti‐CNTN1‐positive patients in our study. Second, enzyme‐linked immunosorbent assay was used to determine the autoantibody positivity, which was not confirmed by cell‐based assay or teased‐nerve immunohistochemistry. Third, because of the retrospective nature of the study, a temporal relationship between the onset of neuropathy and the occurrence of proteinuria was uncertain. Prospective validation is required. Despite these limitations, however, we believe that the simplicity of urinalysis can be a quick method for distinguishing autoimmune nodopathies from CIDP.

In conclusion, proteinuria is a possible biomarker of autoimmune nodopathies associated with autoantibodies targeting CNTN1 or NF155. Autoantibodies against paranodal/nodal molecules, such as CNTN1, NF186, and NF155, should be investigated in patients who present with nephrotic syndrome or mild proteinuria without symptoms. Future prospective studies are needed to clarify the validity of this correlation.

## AUTHOR CONTRIBUTIONS

**Kei Funakoshi:** Conceptualization; methodology; data curation; investigation; formal analysis; writing – original draft. **Norito Kokubun:** Conceptualization; investigation; writing – review and editing; project administration. **Keisuke Suzuki:** Writing – review and editing. **Nobuhiro Yuki:** Conceptualization; writing – review and editing; supervision.

## CONFLICT OF INTEREST STATEMENT

The authors have no relevant financial or nonfinancial interests to disclose.

## Data Availability

The data that support these findings are available from the corresponding author on reasonable request.
